# Urotropin and Helmitol

**Published:** 1906-12

**Authors:** 


UROTROPIN AND HELMITOL.

GUIARD (*Annales des maladies des organes genitourinaries*, October 1, 1905) considers that the efficacy of helmitol is exclusively due to the urotropin which enters into its composition. It has also the same clinical indications, since it is always the urotropin which is its chief active principle. As helmitol is associated with an acid sometimes injurious and of doubtful value, it is better to employ the less complicated drug, urotropin. Again, urotropin will be found to be cheaper than helmitol. Uroptropin is the preferable drug from the standpoint of efficacy, innocuity, and economy. Urotropin must, however, be absolutely perfect. Many of the preparations on the market are of doubtful value, or are positively injurious.—*American Journal of the Medical Sciences*, May, 1906.

